# What Has National Screening Program Changed in Cases with Congenital Hypothyroidism?

**Published:** 2014-06

**Authors:** Şebnem Özgelen, Veysel Nijat Baş, Semra Çetinkaya, Zehra Aycan

**Affiliations:** Clinics of Pediatric Endocrinology, Dr. Sami Ulus Research and Training Hospital of Women’s and Children’s Health and Diseases, Ankara, Turkey

**Keywords:** Congenital Hypothyroidism; Neonatal Screening; Infant; Thyroid Hormones

## Abstract

***Objective:*** Since congenital hypothyroidism (CH) is the most important cause of preventable mental retardation, its screening is important. In this present study, it was aimed to evaluate congenital hypothyroidism cases before and after the initiation of screening program in year 2007 in our country.

***Methods:*** A total of 400 patients diagnosed with CH at our outpatient clinic were retrospectively evaluated. Age of diagnosis, complaint, clinical signs, and etiological distribution were detected and changes in those parameters were evaluated before and after year 2007, which was the initiation date of our national screening program.

***Findings*** ***:*** After year 2007, 70.6% of patients were diagnosed in the first month; 21.2% in 1-3 months; 6.5% in 3-6 months, and no patient was diagnosed after 9 months. Before initiation of the screening program, 48.4% of cases were diagnosed in the first month, the percentage was increased to 62.8% after the program; the increase was significant. While mental retardation was detected in 13.3% of patients before the screening, it was decreased to 4% after initiation of the program. An interesting finding was that age of treatment onset in girls was significantly higher than in boys before the screening; there was no difference between them after initiation of the screening.

***Conclusion:*** In this present study, it was observed that ages of diagnosis and treatment as well as mental retardation rates were significantly decreased in girls after the screening program, but ideal results have not been reached yet, and is expected to be reached in the future.

## Introduction

Congenital hypothyroidism, one of the most important causes of preventable congenital hypothyroidism, is a clinical condition characterized by thyroid hormone deficiency in newborns^[^^1^^,^^2^^]^. If congenital hypothyroidism has not been treated at early stages, it generally causes complications affecting all systems, especially the central nervous system. Although various clinical signs can be observed during the newborn period, no clinical signs might be observed in the early period^[^^1^^-^^5^^]^. Therefore, newborn screening programs are important. Although screening programs have been performed for long time in United States and European countries, it has been started since 2007 in our country. Since Turkey is a region with mild-moderate iodine deficiency, congenital hypothyroidism is encountered and reported higher than its average occurrence in the world^[^^1^^,^^6^^,^^7^^]^.

 Studies regarding changes before and after the screening program are not enough, because the program in this country is quite new when compared with many other countries. It was aimed to perform retrospective clinical and laboratory evaluations of patients diagnosed with congenital hypothyroidism at our outpatient clinic, and also to analyze clinical reflections of the screening program initiated in the year 2007.

## Subjects and Methods

A total of 400 cases with congenital hypo-thyroidism, which were diagnosed and followed up between 1990 and 2010, were evaluated retrospectively. Cases, diagnosed with primary hypothyroidism and isolated hypothyroidism, were included in the study. Other hypophyseal hormone functions were within normal limits in cases diagnosed with the secondary hypothyroidism. Cases followed up for transient hypothyrotropinemia during the newborn period were excluded from the study.

 According to the complaints, some cases had jaundice, prolonged jaundice, coarse facial appearance, goiter, constipation, large tongue, development and growth retardation, mental and motor retardation, large fontanel; whereas some had no complaints, but were diagnosed with hypothyroidism by screening tests. Data for age of diagnosis, year of admission, follow up duration, gender, complaints at admission and clinical signs were collected from the medical records. Patients who applied before 2000 and between 2000 and 2009 were evaluated for each year. Also all parameters were evaluated, and compared with those obtained prior to initiation of the screening program, and after 2007. Moreover, thyroid function tests and imaging (ultrasonography/ scintigraphy) study results of babies at the time of diagnosis were also recorded. TSH values above 100 mIU/L were accepted as 100 mIU/L during the evaluation. All measurable thyroid volumes determined by ultrasonography were calculated by using Delange formula, and they were compared with national data^[^^9^^]^. All variables were analyzed by using descriptive analysis method. Minimum, maximum, mean and standard deviation (SD) values were stated for continuous variables. Dates of admission were given for each year, and then they were grouped and re-evaluated according to cases before and after the year 2007. The results of age-based WISC-R and Denver Intelligence Tests were documented for those for whom these measurements had been performed. Assessments were carried out by a pediatric psychologist.

 Data were statistically analyzed by using SPSS for Windows 18 package program (Statistical Package for Social Science). Homogeneity in a single group was examined by using one sample Kolmogorov Smirnov test. It was observed that all groups were distributed homogenously. Independent sample t test was used in comparisons of measured data between diagnosis groups. Chi Square test was used when comparing categorical variables within themselves, and paired sample t test was used to compare first and final values. Results were expressed as mean±SD (standard deviation) and median for the measured data, and as frequency and percentages for the counted data. The level of significance was accepted as *P*<0.05.

***Findings***

Of 400 cases with congenital hypothyroidism enrolled in the study, 186 (46.5%) were girls and 213 (53.5%) boys. Male/female ratio was 1.15. 225 (56.3%) cases had been admitted in the 17-year period before 2007, initial date of the screening program, and 175 (43.8%) had been admitted in the 3-year period in and after 2007. It was recorded that case numbers were significantly increased after the initiation of congenital hypothyroidism screening.

 The most common admission cause was positive screening test, which was followed by jaundice, prolonged jaundice, mental and motor retardation, growth and development retardation, constipation, coarse facial appearance, goiter and large tongue. Screening test was positive in up to 42.5% of cases. Jaundice and prolonged jaundice were the second most frequent (28%) complaints. While 31.1% of cases were admitted with positives screening test before 2007, the ratio was increased to 57.1% after the screening program; the difference was significant (χ^2^=27.29; *P*<0.001). Jaundice or prolonged jaundice was in the second order among complaints, prolonged jaundice ratio was higher among cases which were admitted before 2007 despite increased jaundice complaint after 2007, but the difference was not statistically significant. Moreover, significant decreased rates were observed in growth and development retardation and mental-motor retardation among cases after the screening test (χ^2^=4.829, *P*=0.046 and χ^2^=10.21, *P*=0.003 respectively) (Table 1). 

 34.3% of the cases were diagnosed in the first 15 days of life, whereas 54.8% were diagnosed within the first month. Before the screening program, 33.3% of cases were diagnosed within the first 15 days, 48.4% were diagnosed within the first 30 days of life. After the screening program was initiated, the rates were increased to 35.4% and 62.8%, respectively. After 2007, there was a significant increase in case diagnosis in the first month of life by using the screening test, but no significant change was determined in case diagnosis in the first 15 days. While 12.8% of our cases were diagnosed after their 12 months of life, the rate was 17.3% before the screening program, and it was significantly decreased to 6.9% after the program (*P*=0.003; χ^2^=9.712). The treatment in 70.6% of cases diagnosed in screening was initiated in the first month of life, 21.2% between 1-3 months of life; 6.5% were diagnosed in 3 and 6 months and 1.8% between 6 and 9 months. There was no patient diagnosed after the 9th month of life. 

 Cases were diagnosed most frequently in the 6th month of life by screening test; they were diagnosed at 6-9 months because of constipation and after 9 months due to mental motor retardation. Although 6 cases had been referred to healthcare units with jaundice in the first 15 days of life, they were diagnosed after the first month of life.

 Ultrasonography and/or scintigraphy showed hypoplasia in 122 (50.4%) cases, thyroid agenesis in 35 (17.2%), hyperplasia in 25 (10.3%), and sublingual thyroid gland in 21 (10.3%) cases. 

 Thyroid function tests (TFTs) were evaluated at admission in all cases. However TSH values of 28 cases were unknown, because their treatment was started in other external healthcare units. Baseline TSH values were between 0-5 mIU/L in 20 (5.0%), 5-10 mIU/L in 19 (4.8%), 10-30 mIU/L in 52 (13.0%), 30-50 mIU/L in 62 (15.5%), and above 50 mIU/L in 219 (54.8) cases. When cases with TSH values of 0-5 mIU/L and cases diagnosed by TRH tests were excluded, mean TSH value was 67.14±32.53 mIU/L.

 Levothyroxine (10-15 μg/kg/d) was immediately started in cases when they were diagnosed with congenital hypothyroidism (serum TSH was ≥10mIU/L or free T4 ≤0.8 ng/dl). 

**Table 1:** Comparison of complaints at admission before and after the screening test

**Main complaint**	**Admission before 2007**	**Admission in and after 2007**	***P-*** **value**
	**n (%)**	**n (%)**	
**Screening**	70 (31.1)	100 (57.1)	<0.001
**Jaundice**	30 (13.3)	34 (19.4)	>0.05
**Prolonged jaundice**	32 (14.2)	16 (9.1)	>0.05
**Mental motor retardation**	30 (13.3)	7 (4.0)	0.003
**Development-growth retardation**	20 (8.9)	6 (3.4)	0.046
**Constipation**	11 (4.9)	5 (2.9)	>0.05
**Coarse facial appearance**	6 (2.7)	1 (0.6)	-
**Goiter**	2 (0.9)	1 (0.6)	-
**Large tongue**	3 (1.3)	--	-
**Large fontanel**	1 (0.4)	1 (0.6)	-
**Other**	8 (3.6)	1 (0.6)	-
**Unknown**	12 (5.3)	3 (1.7)	-
**Total**	225 (100)	175 (100)	

Treatment was initiated within the first 15 days in29.3% of cases, within the first month in 52.8%, and within the first 3 months in 73% of cases. Age of treatment onset before the screening test program was 1.00±2.39 years, whereas it was significantly decreased to the mean of 0.49±1.80 years after year 2007 (*P*=0.019). While treatment was started within 0-15 days in 63 (28%) cases, and within the first month in 103 (45.8%) cases before 2007, the values were increased to 54 (30.9%) and 108 (61.8%) cases after the screening program, respectively.

 Mean age of treatment onset was 1.05±2.65 years in girls, and 0.53±1.59 years in boys; the difference was statistically significant by the independent sample t test (*P*=0.01). Analysis was repeated before the screening program initiated. Before the screening program, girls received the treatment later (*P*=0.005). There was no significant difference between them in 2007 and after (*P*=0.6). Age of treatment onset was not different in boys before 2007 and after (*P*=0.6). Moreover, it was noteworthy that 8 out of 9 patients over 10 years of age were girls.

 Cases on treatment were followed up monthly in the first year of life, then every 3 months after one year of age for growth and development, treatment dose, thyroid function tests and drug side effects. Mean calendar age of our cases was 4.32±4.64 (range 1 month-21 years) decimal year in the last controls, and they were followed up with a mean of 3.74±3.58 (range 1.5 months-19.66 years) decimal year. Treatment was discontinued in 144 cases with the mean treatment discontinuation age of 1.83±1.33 years (0.11-6.41 years). Treatment was re-started in 13 (9%) cases in whom the treatment was discontinued. Time interval between treatment discontinuation and restart was approximately 3.1 months (4-7 months).

 For etiological evaluation of persistent hypothyroidism, hypoplasia was most commonly encountered with 51.7%, the rate of agenesis was 21.7%; dyshormonogenesis 13.8%; ectopic thyroid 10.3%, secondary hypothyroidism 1.5%, and TSH receptor resistance 1%.

## Discussion

Congenital hypothyroidism screening is being performed in many countries at national levels, because CH is one of the most important preventable causes of mental retardation. National CH screening has been initiated since 2007 in our country. We assume that this present study, which has been performed comprehensively and on adequate number of cases, is important to show what changes screening programs can lead to.

 Before the screening program, parents were expected to realize clinical signs of CH and refer to healthcare units. Unclear and nonspecific mental signs of CH during the newborn period caused delayed diagnosis, and persistent changes especially in mental functions^[^^2^^,^^3^^,^^9^^,^^10^^]^. Tarım and Yordam reported in their study conducted on 1000 CH cases between years 1964 and 1989 that the most common complaint was growth retardation (26.7%), which was followed by speech disorders (21.4%), and gait disorders (18.1%). They also reported that complaints were constipation within the first 3 months of life, other causes and large tongue in 4-6 months of age, and growth retardation in cases older than 6 months of age^[^^11^^]^. Karamizadeh et al reported that prolonged jaundice (73%), large anterior fontanel (56%), and wide posterior fontanel (55%) were the most common clinical findings^[^^12^^]^. In our study, the most common cause of admission was positive screening test, and prolonged jaundice which followed this in cases before 2007, whereas jaundice followed it after 2007. 

 It has been realized that age of diagnosis was high before the screening test program. Tarım and Yordam reported that age of diagnosis was in the first month in 3.1%, in 1-3 months of life in 9.4%, in 4-6 months of life in 8.6%, in 7-11 months of life in 9.5%, in 1-2 years of life in 14%, and after 2 years of life in 55.4% of cases^[^^11^^]^. Unachak and Dejkhamron showed in their study from Thailand performed between 1977 and 2000 that 27% of cases were diagnosed in the first 3 months of life; 37.5% within the first year; and 62.5% after 1 year of age^[^^13^^]^. Baserga et al defined the mean age of diagnosis as 23 days (10-57 days) among the admitted patients between 1987 and 2008^[^^14^^]^. When we evaluated data from before and after national screening program, we suggested that the disease was diagnosed in the first month of life (48.4%) especially due to thyroid function tests of patients, who were hospitalized during the newborn period because of different reasons. Moreover, percentage of diagnosed patients within the first 1 month was increased from 48.4% to 62.8% after the year 2007. Considering that some technical and systemic problems have been encountered during the first year of the screening program in Turkey, it can be predicted that age of diagnosis will be decreased in the next years. 

 It is reported that congenital hypothyroidism is generally more common (2:1) in girls^[^^15^^]^. Tarım and Yordam reported in their published study a male/female ratio of 0.86 in 1000 CH cases^[^^11^^]^, Hanukoglu et al reported 0.97^[^^16^^]^, and Karamizadeh et al 1.01^[^^17^^]^. Tamam et al calculated the ratio as 1.19 in their study performed on 182 cases^[^^18^^]^. In our study male/female ratio was calculated as 1.15, which was similar to those in the literature. However, because our study was not designed as an incidence study, incidence studies are required to identify the actual rates in this topic.

 Gender ratios show variability among diagnosed groups in the literature. Hanukoglu et al indicated that agenesis and dysgenesis were more common among girls (61% and 77%, respectively), whereas dyshormonogenesis was lower (35%)^[^^16^^]^. Baserga and Pullano also reported that agenesis was more common among girls^[^^14^^]^. In our study, transient hypothyroidism was more common (62.7%) in boys, whereas ectopic thyroiditis (81%) and agenesis (65.9%) were more common among girls.

 The aim of screening programs is to diagnose CH cases early and to start treatment in the first 15 days of life, so that mental retardation is prevented^[^^1^^-^^4^^]^. Nair et al reported in their study performed on 36 cases in India between 2005 and 2006 that mean age of treatment onset was 3.8±6.1 months although there was no screening program yet. There was no screening program but It was surprising that 61.1% of newborns received early treatment^[^^19^^]^. Baserga and Pullano reported the mean age of treatment onset as 24 (11-58 days)^[^^14^^]^. Şimşek et al found that the mean age of treatment onset was 23±14 days in 11 hypothyroidism patients out of 18606 screened newborns^[^^20^^]^. In our study, mean age of treatment onset was 0.77±1.21 years; the age was decreased significantly after 2007 when the program has been started. Significant decrease in admissions with mental retardation ratio was observed after the screening program because of significant decrease in the treatment age. Moreover, when the ratio was compared according to the gender, it was realized that girls received treatment later than boys. While treatment was started later in girls before the screening program, the ages were equalized between the genders in 2007 and thereafter. Eight out of 9 patients, who were over 10 years old and treated initially, were girls. We think that this is an important finding indicating that girls have started to receive treatment earlier by the screening program. We believe that this gender-related problem may be explained by the condition that boys are considered more important than girls in this country and they are brought to healthcare units earlier.

 Limitation of this current study was a patient based survey and the results may differ from epidemiological studies. However our clinic serves as a referral center for the entire country and patients may also be seen without being referred. Thus, it functions as both a primary and tertiary health care service and we believe that it reflects the general situation of the country.

## Conclusion

This study indicates that although earlier diagnosis and decrease in number of mental retardation cases have been possible after the onset of screening program for CH, the ideal ratios have not been reached yet. Another different and important result is that ages of diagnosis and treatment have been significantly decreased among girls. It is believed that interruptions in the screening program during the early phase have caused that ideal values could not be reached, and the most ideal results will be reached in the near future.

## Authors Contribution:

Ş. Özgelen: concept / design, acquisition of data and data analysis. 

V.N. Baş: concept / design, manuscript preparation, critical revision of the manuscript. 

S. Çetinkaya: concept / design, acquisition of data and interpretation 

Z. Aycan: concept / design, manuscript preparation, data analysis and interpretation and critical revision of the manuscript.

All authors approved the final version of the article. 
